# Connexin 43: An Interface Connecting Neuroinflammation to Depression

**DOI:** 10.3390/molecules28041820

**Published:** 2023-02-15

**Authors:** Hong Jiang, Yi Zhang, Zhen-Zhen Wang, Nai-Hong Chen

**Affiliations:** 1 State Key Laboratory of Bioactive Substances and Functions of Natural Medicines, Institute of Materia Medica & Neuroscience Center, Chinese Academy of Medical, Science and Peking Union Medical College, Beijing 100050, China; 2 Department of Anatomy, School of Chinese Medicine, Beijing University of Chinese Medicine, Beijing 102488, China

**Keywords:** depression, connexin 43, neuroinflammation, antidepressant target, glial cells, blood-brain barrier

## Abstract

Major depressive disorder (MDD) is a leading chronic mental illness worldwide, characterized by anhedonia, pessimism and even suicidal thoughts. Connexin 43 (Cx43), mainly distributed in astrocytes of the brain, is by far the most widely and ubiquitously expressed connexin in almost all vital organs. Cx43 forms gap junction channels in the brain, which mediate energy exchange and effectively maintain physiological homeostasis. Increasing evidence suggests the crucial role of Cx43 in the pathogenesis of MDD. Neuroinflammation is one of the most common pathological features of the central nervous system dysfunctions. Inflammatory factors are abnormally elevated in patients with depression and are closely related to nearly all links of depression. After activating the inflammatory pathway in the brain, the release and uptake of glutamate and adenosine triphosphate, through Cx43 in the synaptic cleft, would be affected. In this review, we have summarized the association between Cx43 and neuroinflammation, the cornerstones linking inflammation and depression, and Cx43 abnormalities in depression. We also discuss the significant association of Cx43 in inflammation and depression, which will help to explore new antidepressant drug targets.

## 1. Introduction

Major depressive disorder (MDD) is a severe mood disorder characterized by chronic depression and cognitive impairment [1]. This mental illness poses a significant economic burden, accounting for 10.3% of the total burden of disease [2]. Currently, only about one-third of people with MDD are treated with antidepressants that produce complete remission. So far, there are no biomarkers to guide physicians in choosing the best treatment for an individual [3]. Genetic and environmental interactions both contribute significantly to depression. The neuropathological effects of MDD are related to various influencing factors, including monoamines, glutamate receptors, inflammatory cytokines, hypothalamic–pituitary–adrenal axis, epigenetic modifications, and neurotrophic factors [2].

Gap junction channels (GJCs) are specialized membrane domains that allow for intercellular ion exchange and members of the metabolome <1000 Da [4]. Connexin is a protein widely distributed in the body. Six connexin subunits form a connexon [5]. A GJC is formed by a pair of connexons in adjacent cell membranes, while uncoupled connexons exist as hemichannels (HCs) [6]. Gap junctions allow for communication between cells through ion exchange and small molecules acting as secondary messengers, such as calcium ions (Ca^2+^), nicotinamide adenine dinucleotide, 1,4,5-trisphosphate receptors (IP3Rs), adenosine triphosphate (ATP), glutamate, and glucose [7]. Moreover, uncoupled connexins serve as plasma membrane HCs involved in releasing small signaling molecules and glial transmitters to the extracellular environment [8]. Notably, connexin 43 (Cx43) is identified as a significant member of the connexin family, with a half-life ranging from 1 to 3 hours [9] and a predicted weight of 43 kDa [10].

Cx43 interacts with cytoskeletal elements tubulin and membrane-associated guanylate kinase proteins, such as zonula occludens-1 (ZO-1). It is phosphorylated by various kinases, such as tyrosine protein kinase, encoded by the sarcoma gene and protein kinase C (PKC) [11,12]. These interactions regulate the formation and stability of Cx43 gap junctions [12]. For example, ZO-1 interacts with Cx43 to negatively regulate gap junction size [13]. GJCs are highly implicated in regulating nerve cell growth, differentiation and physiological function through metabolic coupling involved in the intercellular material exchange, electrically coupled electrical signal transmission, and intercellular information transmission [14]. The opening of HCs under stress conditions is beneficial to increase the fitness of cells in the short term. Still, long-term activation might cause damage to cells, thereby causing the disease [6].

Neuroinflammation is the localized inflammation that occurs in the central nervous system (CNS) and plays a part in inhibiting infection, eliminating pathogens and erroneously folded proteins. The release of inflammatory mediators, increase in vascular permeability, leukocyte infiltration, and glial cell activation are the four main characteristics of neuroinflammation [15,16]. To date, it has been demonstrated that brain inflammation is closely related to a variety of neuropsychiatric disorders, including Alzheimer’s disease (AD), Parkinson’s disease (PD), epilepsy, and depression [17,18,19,20].

As complex, innate, immune neuroinflammation varies from the inflammation in peripheral tissues [21], the communication in the CNS may cause impaired neurogenesis and neural plasticity. CNS is a so-called immune-privileged region due to the blood-brain barrier (BBB), with unique anatomy and physiology [22]. When damage occurs to surrounding tissue, white blood cells are drawn through the bloodstream to the injury site [23,24]. Infiltration of leukocytes and pro-inflammatory mediators, such as tumor necrosis factor-α (TNF-α), interleukin-1β (IL-1β), and interleukin-6 (IL-6), released promote the increase in the permeability of the BBB, causing the entering of the blood cells and immune cells to the CNS in the direction of the blood flow [23]. Then, the immune cells and blood cells transform into reactive microglia, releasing pro-inflammatory cytokines and causing the activation of the microglia and astrocytes [25]. Once microglia and astrocytes are activated, a cascade of neuroinflammation is also activated, and inflammation spreads from the periphery to the center.

Changes in Cx43 of astrocytes and inflammatory signatures can appear in different neuropsychiatric diseases (Table 1). It is worth emphasizing that Cx43 dysfunction in astrocytes is an important pathological feature of depression. As a newly discovered consequence, the inactivation of the astrocyte Cx43 gene enhances the antidepressant effect of fluoxetine [26]. Evidence shows that neuroinflammation is one of the most common pathological features of CNS dysfunctions [27]. When some inflammatory pathways in the brain are activated, the release of inflammatory factors interferes with the opening and closing of Cx43 GJCs and HCs, thus affecting the normal function of Cx43 [28]. To some extent, the activation of the inflammatory pathways determines Cx43 exercising normal functions in the brain. Thus, Cx43 might act as an intermediate link between neuroinflammation and depression. In the present review, we discuss the potential relationship and summarizes the latest views on the role of Cx43 in neuroinflammation and depression-like behaviors, aiming to explore more targets and new ideas for the treatment of depression.

## 2. Association between Cx43 and Inflammation

**Figure 1:**
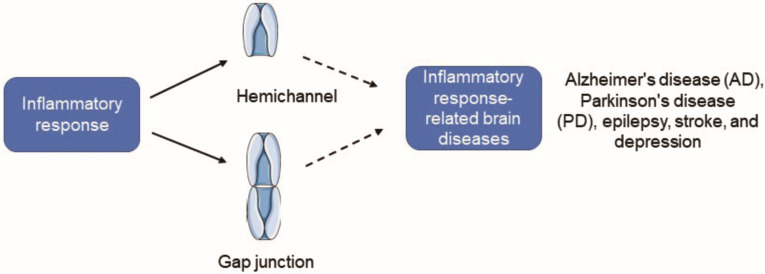
Cx43 HC activity is increased and Cx43 GJIC is inhibited under inflammatory conditions, and it will induce the occurrence of some inflammation-related neuropsychiatric diseases. Cx43, Connexin43; HC, hemichannel; GJC, gap junction channel; GJIC, gap junction intercellular communication.

A transient inflammatory response exerts a protective homeostatic role, whereas a durable tissue response maintains unregulated chronic inflammation, which is thought to be a major cause of disease progression [40]. Delving into the CNS, cellular crosstalk between astrocytes and microglia forms a positive feedback loop, leading to the dysregulation and self-amplification of the inflammatory response [41]. Chronic neuroinflammation, accompanied by Cx43 disturbance, is a characteristic of neuropsychiatric diseases, although the underlying signaling mechanisms remain poorly understood (Figure 1). Hence, therapeutic development of Cx43 will undoubtedly become a promising target for anti-inflammatory therapy, which provides a feasible research direction for the therapeutic methods.

### 2.1. Cx43 Gap Junction Channels and Cx43 Hemichannels

The HCs of two adjacent cells combine to form a GJC, which allows the passive diffusion of small molecules between the cells [42]. Stimulation of microglia by LPS triggers a switch between Cx43 channel properties manifested in the inhibition of Cx43 GJCs in astrocytes [43]. Similar phenomena are still confirmed in experiments in vivo. From the observation of whole-cell patch-clamp recordings on green fluorescent protein (GFP)-positive astrocytes in acute brain slices from glial fibrillary acidic protein-GFP mice, following Staphylococcus aureus infection in the striatum, astrocyte gap junctional intercellular communication (GJIC) is markedly attenuated in regions near the brain abscess [44]. With increasing distance from the proper site of the abscess, gap junctional communication in astrocytes gradually increases, even to the levels typical of uninfected brains [44]. However, there is no unified conclusion on the mechanism of neuroinflammation inhibiting Cx43 GJIC, which mainly focuses on mitogen-activated protein kinase (MAPK) signaling pathway activation, and changes in Cx43 phosphorylation, synthesis, as well as degradation [45,46,47].

From the available conclusions, HC opening appears to play a regulatory role in diverse brain diseases. A short-term opening increases cell fitness, while a long-term opening causes damage to cells [6]. The permeability of Cx43 HC to bromoethane (EtBr), a cellular uptake of the non-fluorescent permeable tracer, is a significant indicator reflecting the activity of HC. When an inflammatory abscess occurs, the increase in inflammation near the abscess is accompanied by increased activity of astrocyte HCs, mainly manifested as increased EtBr uptake [44]. Adolescent injection of ethanol in rats increases the opening of Cx43 HCs in hippocampal astrocytes through the inflammatory pathway [48]. The opening of HCs affects the interaction of glial cells, releases a large number of toxic substances, such as ATP and glutamate, promotes the spread of damage, aggravates cell death, and affects neurological function [49]. The mechanism by which neuroinflammation increases the activity of Cx43 HC is complex and not clear. Moreover, a growing body of evidence states that it is related to the changes in extracellular Ca^2+^ levels, disorders of oxygen and glucose metabolism, and activation of the MAPK and N-methyl-D-aspartate receptor (NMDAR) signaling pathways during neuroinflammation [50,51,52].

### 2.2. Function of Cx43 in Neuroinflammation

#### 2.2.1. Astrocytes

Cell coupling is a fundamental biological property of astrocytes. Disruption of astrocyte syncytium coupling efficiency is followed by higher astrocyte mortality and impaired neuronal function. Cx43 upregulation is identified in rotenone-stimulated cultured astrocytes and the striatum of the PD model [32]. Astrocytes Cx30 and Cx43 are increased near amyloid β-protein (Aβ) plaques in the AD mouse model [53]. Neurosurgical specimens from epilepsy patients often show marked reactive gliosis, with astrocytic Cx30 and Cx43 mRNAs expressing several times more than neuronal Cx36 and Cx45 mRNAs [32]. Beyond that, allogeneic cell-to-cell communication between astrocytes and glioblastoma cells via gap junctions, and glioma growth is reduced when Cx43 is overexpressed [54]. In ischemia, blocking autophagic degradation of Cx43 favors the transition of astrocytes from a pro-inflammatory to an anti-inflammatory state [55].

ATP and glutamate play critical roles in neuroinflammation. ATP is a crucial regulator of the nucleotide-binding domain and leucine-rich repeat protein 3 (NLRP_3_) inflammasome activation in the induction of neuropathic pain. Peripheral nerve injury may induce the opening of Cx43 HCs in spinal astrocytes to release ATP, thereby activating the NLRP_3_ inflammasome [56]. Astrocytes protect against glutamate-induced oxidative stress and excitotoxicity and regulate synaptic processes [57]. In contrast, abnormal Cx43 expression directly causes a glutamate tripartite synapse imbalance and the physiological state of the associated cells. Released ATP and glutamate, following activation of Cx43 HCs, mediate neuronal death [58]. Conversely, neurons upregulate astrocyte gap junction communication and Cx43 expression [59].

Astrocytes can also communicate with other cell types by forming a GJC. For instance, astrocytes couple to oligodendrocytes to form glial syncytia via Cx43 GJCs. Early loss of astrocytic Cx43 may promote oligodendrocyte apoptosis by disrupting glial syncytia [60]. Alongside that, Cx43 gap coupling exists between astrocytes and endothelial cells, which contributes to the integrity of the BBB [61,62]. Astrocyte Cx43 inactivation leads to an abnormal recruitment of B and T cells, macrophages, and neutrophils in the brain and across the BBB. Moreover, the association between Cx43 expressed in astrocytes and neuroinflammation is far more complicated than the current literature reports. Knockout of Cx43 in astrocytes induces long-term loss of neuroplasticity in mice [63]. Interestingly, acute experimental autoimmune encephalomyelitis does not worsen in Cx43 knockout mice, indicating that leukocyte recruitment caused by Cx43 deletion may help reduce inflammation [64].

#### 2.2.2. Microglia

Activation of microglia, a major source of inflammatory mediators and free radicals in the brain, is associated with changes in behavior and modulation of neural excitability [65,66,67]. In resting microglia, connexins are expressed at low or even undetectable levels [68]. Cx43 is an essential protein subunit of gap junctions between microglia, only expressed in activated microglia [2,69]. INF-γ plus TNF-α treatment significantly increases the incidence of dye conjugation and Cx43 levels [69]. Moreover, activation of microglia is normally accompanied by the release of inflammatory factors, such as IL-β, TNF-α and IL-6. Elevated levels of TNF are especially considered as a hallmark of neurodegenerative diseases [70].

Robust functional interactions exist between astrocyte and microglia cell types [71,72]. Proinflammatory cytokines released by activated microglia trigger opposing regulations of Cx43 function in astrocytes; astrocyte gap junction coupling is reduced [73]. By contrast, astrocytic Cx43 HC opening increases, impairing glial cell interactions [43,49]. Concerning the specific functional links, phosphorylation of substrates is a feasible research direction. As we know, TNF-α affects PKC activation, leading to the depolarization of astrocyte resting membrane potential [74]. In addition, unitary conductance and coupling conductance of Cx43 channels have been proven to be dependent upon phosphorylation by multiple kinases, such as PKC and PKA [75].

Some studies have also explored the changes of Cx43 in microglia under different states. For instance, astrocytes interfere with the number of microglia and the secretion of cytokines [76]. The Ca^2+^ signaling between astrocytes and microglia, mediated by ATP, even induces the death of microglia [77]. BV2 cells are immortalized cell lines derived from mouse microglia. In investigating the multiple important roles underlying ATP release from neural cells, Cx43 HCs in BV2 microglia open in the presence of cyclic adenosine diphosphate (ADP) ribose signaling inhibition [78]. After that, production of Cx43-dependent ATP release induces the activation and death of BV2 microglia cells [78]. Moreover, Aβ is shown to increase the expression of Cx43 on the cell surface of microglia, which forms functional HCs, allowing for the release of ATP and glutamate to the extracellular milieu [68].

#### 2.2.3. Immune Cells

In terms of current studies, immune cells serve a pivotal role in the development of inflammation. When peripheral cytokines disrupt the integrity of the BBB, leukocytes, such as activated CD4+T cells, can infiltrate the CNS, participating in neuroinflammatory feedback [79]. Meanwhile, Cx43, contributing to maintaining direct intercellular communication between lymphocytes, is widely expressed in immune cells, such as monocytes, B as well as T cells [80]. Cx43 is a novel astroglial factor that promotes immune dormancy in the brain [81,82]. A recent study demonstrated that astroglial Cx43 controls the recruitment of immune cells by setting the activation state of the cerebral endothelium [83]. As to how astroglial Cx43 is involved in the regulation of the immune recruitment, studies have suggested that astrocyte functional Cx43 HCs tune synaptic transmission of transient glutamate, which is shown to control the expression of neuroinflammatory molecules and major histocompatibility complex (MHC) class II molecules [83]. Of note is that MHC class II molecules play a critical role in the initiation of the antigen-specific immune response [84].

Cx43 GJCs and HCs serve a critical role in cell-to-cell communication. Cx43 GJIC can be established between T lymphocytes and macrophages. Some monocytes/macrophages aggregate at the sites of inflammatory responses to transiently form Cx43 GJCs, thereby coordinating the metabolism and electrical conduction of the cell communities [85]. Meanwhile, Cx43 is considered to contribute in the leukocyte–endothelial cell communication during cell migration. Activated neutrophils release ATP via Cx43 and regulate adenosine-dependent endothelial cell function [86]. Moreover, brain metastatic cancer cells also utilize cancer astroglial GJCs to assemble the transfer of the second messenger to the astrocytes in the brain, activating inflammatory pathways and inducing the production of inflammatory cytokines, such as IFN-α [87].

Various methods that could regulate the function of GJCs and HCs are used to study the role of connexins in immune-related diseases. For instance, interfering with the activity of the cell surface Cx43 HCs abrogates T-cell proliferation and activation [88]. The blockade of Cx43 channels in T lymphocytes results in a decreased accumulation of circulating inflammatory cells in hypertensive patients and spontaneously hypertensive rats [89]. Besides, monocyte–endothelial adhesion is a major initiator of inflammatory vascular disease. When propofol inhibits the expression of Cx43 in monocytes, ZO-1 decreases with the down-regulation of Cx43. Down-regulated Cx43 modulates signaling pathways that alleviate monocyte adhesion, and thus reduces the inflammation induced [90].

#### 2.2.4. Endothelial Cells

The BBB, preventing over 98% of small molecules and antibodies from entering the parenchyma, is composed of endothelial cells, pericytes expressing Cx37, Cx40, and Cx43, and astrocytes expressing Cx26, Cx30, and Cx43 [91]. The major function of the BBB is to transmit biochemical signals in the blood to the brain, which requires a plethora of signaling cascades. BBB failure correlates with neurological diseases such as stroke and AD, in which endothelial Cx43 serves a role [92]. Experiments demonstrate that endothelial Cx43 HCs contribute to the induction of cell death and inflammatory processes resulting in endothelial cell damage [93]. Cx43 is even detected at the cellular interface between endothelial and monocyte/macrophage cells in the BBB model [85].

A recent experiment indicates that in mice with elevated serum gonadotropins after ovariectomy, alterations in reproductive hormone signaling pathways lead to an increased expression of Cx43 in endothelial cells and mediate the BBB permeability change [94]. Thus, we propose that Cx43 is a key part in the regulation of BBB permeability. Interaction with tight junction proteins contributes to stabilize the Cx43 junctional complex between epithelial and endothelial cells. Junctional instability abrogates the barrier function of epithelial and endothelial cells, promoting leukocyte migration to inflamed tissues [95]. Cx43 gap junctions also instigate the BBB breakdown by regulating ZO-1 localization and limiting tight junction formation and interaction [96].

Endothelial cells form Cx43 couplings with other cell types. Pericytes are vascular wall cells, embedded within the same basement membrane as endothelial cells. Crosstalk between endothelial cells and pericytes relies on secreted paracrine factors, the formation of adhesion plaques and gap junctions [97]. Cx43 is one of the major gap junction proteins which participate in direct contact formation of endothelial cells and pericytes [97]. Dependent crosstalk of Cx43 gap junctions in pericytes and endothelial cells contributes to the BBB integrity [97]. Alongside that, limited astrocyte–endothelial Cx43 gap junction coupling exhibits in cell co-cultures [98]. Deletion of astrocytic Cx43 results in aberrant expression of astrocyte perivascular endfeet markers. These alterations weaken the BBB, which may promote immune cell infiltration into the brain parenchyma and propagating of inflammatory responses [99]. In the induction of inflammation, especially neuroinflammation, the signaling cascade involved in endothelial cell Cx43, regulating the BBB, is complex. For instance, Cx43 is downregulated at the sites of inflammation in the CNS, while astrocyte gap junctions associated with BBB disruption do not play a modulating role in acute experimental autoimmune encephalomyelitis [100].

## 3. The Cornerstones Linking Inflammation and Depression

As one of major disabling diseases, the etiology of MDD is unknown. It should be recognized that a complex relationship exists between depression and inflammation, supported by clinical data and related experimental results [101]. Inflammatory disorders of the peripheral and central nervous systems contribute significantly to mood disturbances, especially among young adults [102]. In turn, accumulating evidence shows that stress triggers neuroinflammation, leading to anxiety and depression-like behaviors, destroying the neuroprotective mechanism in the brain, inhibiting the expression of tubulin and the extension of axons and dendrites, and impairing neuroplasticity [103]. The vital link between depression and inflammation will be discussed in diversiform ways in this paragraph.

### 3.1. Clinical Evidence

#### 3.1.1. Increased Inflammatory Response in Patients with Depression

Cytokines are small proteins with pro- or anti-inflammatory effects. Patients with depression exhibit an increased expression of proinflammatory cytokines and their receptors in the peripheral blood and cerebrospinal fluid (CSF), especially IL-1β, IL-6 and TNF-α, as the cardinal feature of the inflammatory responses [104,105]. Miller and coauthors previously presented data showing that poor children show greater levels of depression and anxiety than children with a better economic status, along with blood markers of inflammation like C-reactive protein and cytokines [106]. Moreover, patients with higher levels of inflammation, measured through C-reactive proteins, have less response to standard of care treatment for MDD, suggesting that immune-inflammatory events play a role in the development of treatment resistance [107].

When discussing the improvement of inflammation by antidepressants, the effects of antidepressants on the modulation of cytokines remain unclear. Based on a large number of animal experiments and clinical data, it is generally considered that antidepressants tend to promote anti-inflammatory responses. However, in other studies, antidepressants induced the production of IL-6 and TNF [108]. Moreover, the levels of pro-inflammatory cytokines are increased in patients with treatment-resistant depression (TRD), suggesting that treatment response is inversely correlated with pro-inflammatory cytokine levels [108]. In addition to sources of heterogeneity, including baseline inflammation, smoking, and antidepressant drug classes, more relevant pathological mechanisms and mechanism channels need to be studied to explain this inconsistency between treatment effect and an increase or decrease of proinflammatory factors.

#### 3.1.2. High Comorbidity Rate of Depression and Multiple Immune-Based Diseases

A growing body of evidence highlights that depression has a high comorbidity with an increased risk of multiple immunologically underlying diseases, including rheumatoid arthritis, cardiovascular disease, certain cancers, and neurodegenerative diseases [109]. Therefore, numerous clinical studies have consistently linked somatic disorders involving inflammatory pathophysiological mechanisms with an increased risk of depression. Detection of obesity is often accompanied by the use of body mass index, which is also positively correlated with high levels of IL-6 [110]. Another study has found that obesity in late adolescence is associated with an elevated risk of MDD in adulthood, and MDD that appears in early adolescence is related to an increased risk of late-onset obesity [110]. The course of rheumatoid arthritis is relatively long and prone to recurrent attacks, which aggravates the mental burden of patients and causes depression. In patients with rheumatoid arthritis, circulating levels of cytokines, such as TNFα, are elevated [65]. In addition, administration of TNF-α to healthy subjects results in the development of depressed mood [65]. In anti-TNF-α therapy in patients with rheumatoid arthritis, functional magnetic resonance imaging shows that anti-TNF-α treatment rapidly alters the brain’s response to pain peripheral stimuli and improves patients’ sense of well-being [65].

#### 3.1.3. Inflammatory Factors Induce Depression

Enhanced coupling between neurological disorders and inflammatory processes seems to be bidirectional. Not only does inflammation mediate depression risk and neural progression, but higher levels of inflammation appear to increase the risk of new, onset depression. Excessive inflammatory cytokine activity perturbs multiple neuronal functions affecting neurotransmitter metabolism and neuroplasticity [108,111]. A recent hypothesis suggests that proinflammatory cytokines may contribute to the development of MDD through the activation of indoleamine 2,3-dioxygenase [112]. The enzyme indoleamine 2,3-dioxygenase metabolizes tryptophan into quinolinic acid and neurotoxic compounds, 3-hydroxykyurenin [112]. This would lead to the depletion of locally stored tryptophan, which is necessary for serotonin synthesis and induces depression [112]. Inflammatory cytokines are also pro-inflammatory agents to treat certain medical conditions, such as hepatitis C or malignant melanoma [20]. However, long-term exposure to cytokines also leads to dramatic changes in human behavior. For instance, about 40% of patients with hepatitis C infection or certain cancers, treated with interferon-α, experience depressive symptoms after starting treatment [101].

### 3.2. Experimental Evidence

A chronic mild stress depression model, commonly used as the animal model of depression, has the greatest validity and translational potential with a similar phenotype, risk factors, and response to routine treatments used in MDD patients [113]. Various experiments show that chronic unpredictable mild stress leads to increased inflammation-related symptoms when induced in an animal model of depression [114,115,116], suggesting that inflammation may be an intermediate link between the mechanisms of depression. In another study, lipopolysaccharide (LPS) stimulation triggered central inflammation by mimicking acute inflammatory events, resulting in depressive-like behaviors [117].

## 4. Cx43 Abnormalities and Dysfunction in Depression

Postmortem studies have found down-regulated Cx43 gene and protein expression in the frontal cortex [118], hippocampus [119], locus coeruleus [120], dorsomedial nucleus of the thalamus [121], and caudate nucleus [122] in MDD patients. Pyrosequencing analysis detects no significant differences in GJA-1 (Connexin43) genomic methylation status between patients and healthy individuals [123]. Cx43 is significantly down-regulated in depressed patients and animal models of depression [124]. Overall, insufficient expression of Cx43 or modification of dysfunction might contribute to pathological changes in MDD.

Cx43 abnormalities and dysfunction exist in depression. Exposure to chronic unpredictable stress (CUS) over a few weeks, immunostaining of Cx43 and myelin basic protein is reduced in the prelimbic and orbitofrontal cortices of rats, implicating consistent mechanisms of astrocyte connexin alterations and myelin morphological disturbances [122]. CUS suppresses the permeability of the gap junction and significantly expands the gap width [125]. In mice treated with a chronic socially frustrating stress model, Cx43 expression was down-regulated in the medial prefrontal cortex and hippocampus, inhibiting local neuronal activity and inducing depressive behaviors [126]. Remarkably, long-term exposure to corticosterone induced anxiety- and depression-like abnormalities in mice, that also increased the amounts of phosphorylated forms of Cx43 in the hippocampus [127].

Compared with wild-type mice, the administration of fluoxetine in Cx43 knockout mice enhanced the antidepressant effect in the tail suspension test [26]. Thus, we propose that CX43 affects behaviors in depression. For instance, depletion of cortical or hippocampal glial cells accompanied by disturbances in Cx43 expression leads to neuronal dysfunction, causing the expression of depression-like symptoms [125,128]. And overexpression of Cx43 in the hippocampus suppresses depression-like behaviors and increases neuronal activity [126]. Some researchers have investigated the role of Cx43 in depression by blocking Cx43 with drugs. In the rat prefrontal cortex, alcohol preference and intake increased with GJC blockade [129]. The sucrose preference experiment is an important behavioral method for judging whether animals are depressed. The ratio of sucrose volume to total sucrose volume and water consumption was significantly reduced in treated animals bilaterally receiving carbenoxolone, a widely used gap junction blocker [125]. Overall, the disturbance of Cx43 affects the induction of depressive behavior to some extent.

## 5. Cx43 as a Mediator of Neuroinflammation and Depression

Depressed patients are often accompanied by neuroinflammation, which is both a cause and a consequence of depression, forming a feedback pathway. Cx43 expression plays an important role in the pathophysiology of depression. This section mainly discusses the changes of Cx43 in the brain of patients with depression, associated with inflammation, the possible role of Cx43 in treatment-resistant depression, the possible changes of Cx43 during the application of antidepressant and anti-inflammatory drugs, and the related pathogenesis.

### 5.1. Regulation of Cx43 by Inflammatory Cytokines in Depression

Cytokines are biologically active, acting on limited receptors per cell and subsequently activating giant genes to amplify their effects [130]. A large number of clinical and experimental data shows that inflammatory factors, which can be suppressed by antidepressants, are abnormally elevated in MDD patients [131,132]. When the inflammatory pathway is activated to induce depression, microglia release inflammatory factors to impact astrocyte Cx43 and the CNS environment. Activated astrocytes also secrete TNF-α, IL-1β, as well as IL-6 [67].

Inflammatory cytokines conduce to glial cell metabolism and barrier integrity. Tumor necrosis factor receptors 1 (TNFR1) and 2 (TNFR2) are the two receptors for TNF-α, mediating the effects of TNF-α in neurogenesis [133]. The CA1 region is located in the hippocampus of the brain. TNF-α released by microglial reduces CA1 pyramidal cell dendrites of hippocampal slice cultures, and modulates excitatory synaptic connections [134]. Anti-TNF-α treatment improves patient well-being before significant changes in tissue inflammation, in patients with rheumatoid arthritis [65]. Reactive astrocyte IL-6 specifically destroys BBB function by stimulating the proteasomal degradation of ZO-1 [135]. Infiltration of IL-6 into the brain parenchyma is followed by expression of depression-like behaviors [136]. IL-1β exacerbates BBB disruption by inducing astrocytic chemotaxis of C-C motif ligand 2 (CCL2), C–C motif ligand 20 (CCL20), and C–X–C motif chemokine ligand 2 (CXCL2) [137]. CCL2, CCL20, and CXCL2 are chemokines inducing the directional migration of cells. ATP is disturbed in the presence of inflammation and depression. IL-1β upregulates the human astrocyte purinergic P_2_X_7_ receptors (P_2_X_7_Rs) in response to changes in extracellular ADP and ATP concentrations [137].

Studies show that in astrocytes co-cultured with microglia, microglia inhibit Cx43 gap junctions by releasing inflammatory factors [43,60,75]. However, the activity of Cx43 HC is enhanced, which is also accompanied by the enhanced exchange between cells and the extracellular environment [43]. It is proposed that intercellular GJCs control glutamate homeostasis and maintain intercellular Ca^2+^ wave propagation, while they can be impaired by IL-1β and TNF-α [73]. The proinflammatory cytokine IL-1β is individually applied to inhibit the GJIC of Cx43 weakly, while TNF-α, IFN-γ, or IL-6 have no effect [43]. In contrast, TNF-α and IL-1β are individually potent activators of Cx43 HC. Similar treatments result in the opening of Cx43 HC in the fresh astrocytes and brain slices [138]. Inflammatory factors are closely related to every link of depression, and the regulation of Cx43 by inflammatory factors may be an intermediate link in the pathogenesis of depression.

### 5.2. Cx43 in Treatment-Resistant Depression and Neuroinflammation

Long-term social affiliation in mice leads to depression-like behaviors. In contrast, adaptive mice resist the development of depression, with preexisting differences in the sensitivity of individual immune systems determining vulnerability or resilience to social stress [139]. Depression causes a severe decline in the quality of life and is prone to relapse. Despite various treatment methods and drugs for depression being applied clinically, only a small proportion of patients achieve remission [140]. Treatment of depression is significantly more difficult supposing a patient does not respond satisfactorily to adequate dose and duration antidepressant treatment trials, usually referred to as TRD [141]. The proportion of MDD patients classified as TRD is as high as 15–30% [142]. Inflammatory processes are involved in the pathophysiology of TRD, and enhanced baseline levels of inflammatory markers predict poorer antidepressant efficacy in depressed patients [113]. The elevated inflammatory factors in the patients’ bodies are an example. Elevated pro-inflammatory factors reduce 5-hydroxytryptamine (5-HT) content in the synaptic cleft, increase glutamate accumulation, and induce NF-κB expression to hinder the effectiveness of traditional antidepressants [143,144,145].

The pro-inflammatory factor IL-1β is an example. The exchange of information between Cx43 channels and the release of transmitters from HCs are regulated by IL-1β. In elderly patients with depression, IL-1β is abnormally elevated in patients who are resistant to antidepressants [146]. In animal experiments, IL-1β was injected into the lateral ventricle. In addition to neuroinflammation, animals exhibited signs of depressive behavior [147,148]. Il-1β causes fluoxetine resistance in the animal model of epilepsy-induced depression, with subsequent presynaptic 5-HT1A receptor up-regulation. A combination of fluoxetine and interleukin-1 receptor antagonist therapy completely abolished all features of epilepsy-related depressive abnormalities in the setting of monotherapy resistance [149].

Thus, Cx43 might act as a hub between TRD and neuroinflammation. Due to the lack of sufficient clinical and experimental data to support this, the specific mechanism and difference between curable patients and healthy people are unclear, therefore various influencing factors should be taken into consideration in further studies. However, it is undeniable that the expression of Cx43 GJCs and HCs is an essential target in the treatment of MDD and TRD.

### 5.3. Effects of Antidepressants and Anti-Inflammatory Drugs on Cx43

#### 5.3.1. Antidepressants

Antidepressants refer to a group of psychoactive drugs used to treat mental illnesses with prominent symptoms of depression, clinically mainly divided into tricyclic and tetracyclic antidepressants (TCAs), 5-HT and adrenaline reuptake inhibitors (SNRIs), and 5-HT reuptake inhibitors (SSRIs) [150]. Depressed brains show down-regulation of Cx43, whereas antidepressant treatment favors up-regulation of Cx43. Fluoxetine reverses the reduction of Cx43 in rats subjected to CUS in the prefrontal cortex [151]. Furthermore, the antidepressants fluoxetine and duloxetine are used in CUS-treated rats to reverse the decreased intercellular coupling of Cx43 and the increased GJC width of the ultrastructural alterations [125]. When cortical astrocytes are cultured with antidepressants, including amitriptyline, clomipramine, and fluvoxamine, the expression of Cx43 is up-regulated [152].

LPS-stimulated microglia co-cultured with astrocytes, the proinflammatory cytokines TNF-α and IL-1β released by microglia, activate astrocyte Cx43 HCs with additive effects [43]. Furthermore, seven monoamine antidepressants tested (fluoxetine, amitriptyline, imipramine, reboxetine, paroxetine, duloxetine, and venlafaxine) can inhibit LPS-induced enhanced astrocyte HC activity [153]. Among them, venlafaxine has the lowest inhibitory effect on LPS-induced HC activities and is tested to increase TNF-α levels [154]. Generally speaking, however, antidepressants, particularly SSRIs, reduce the production of pro-inflammatory cytokines like TNF-α, and increase anti-inflammatory cytokines like IL-10 [155]. Ketamine, a novel rapid-acting antidepressant, reduces serum and cerebrospinal fluid IL-6, and serum IL-1β and TNF-α, while treating depression [156]. Ketamine exhibits anti-inflammatory properties and modulates the brain’s innate immune response [157]. Acute treatment with therapeutically relevant concentrations of ketamine inhibited the activity of Cx43 HCs, but not GJCs, possibly by inhibiting the release of TNF-α and IL-1β [6]. Because of the different results of the drug effect on Cx43, we can guess that the receptors and triggering pathways of different antidepressant drugs are not the same due to the specific mechanism. In addition, due to the differences in dose and time of administration, and other more complex situations, antidepressant efficacy and drug resistance appear in clinical and experimental studies.

#### 5.3.2. Anti-Inflammatory Drugs 

Some drugs improve depression-like behavior while working as an anti-inflammatory. Gallic acid from Terminalia Bellirica fruit inhibits CMS-induced serum corticosterone levels in mice treated with CMS, and exerts antidepressant-like effects in behavioral observations [158]. Likewise, Ginsenoside Rg1 is one of the constituents extracted from ginseng, with antidepressant and anti-inflammatory neuroprotective properties [159]. Ginsenoside Rg1 attenuates neuroinflammation by inhibiting Cx43 ubiquitination, thereby reducing depression [160]. Similarly, minocycline, an antibiotic, reduces the proinflammatory cytokines from activated microglia and relieves depression [161].

Celecoxib is a cyclooxygenase-2 (COX-2) inhibitor proven to cross the BBB [162]. Celecoxib could alleviate depression-like behavior in mice [163]. Besides, the topical application, celecoxib may effectively inhibit rat tongue cancer by regulating the expression of Cx43 proteins [164]. Neuroinflammation and COX-2 up-regulation are implicated in the pathogenesis of degenerative brain diseases, and COX-2 is induced in depressive responses [165]. COX-2 inhibitors selectively regulate monoamine metabolism in the rat cortex. When co-administered with the SSRI antidepressant fluoxetine, celecoxib enhances the release of dopamine and serotonin, synergizing the therapeutic effect of antidepressants [161]. However, whether celecoxib and other anti-inflammatory drugs regulate depression through the BBB and Cx43 still needs further research.

Depressive symptoms often improve after immunosuppressive drug treatment in people with inflammatory diseases. Adjunctive antidepressants with anti-inflammatory drugs appear to enhance the effectiveness of antidepressants. Non-steroidal anti-inflammatory drugs are used as add-ons to conventional antidepressants in most studies [110]. Thus, anti-inflammatory drugs play a certain antidepressant effect or synergistic antidepressant effect.

### 5.4. Pathogenesis Associated with Cx43 and Neuroinflammation

Together with presynaptic and postsynaptic neurons, astrocytes establish “tripartite synapses” and locally release paracrine substances called “glial transmitters”, such as ATP and glutamate, to sense neurotransmission [48]. Activation of Cx43 HCs in astrocytes releases glutamate and ATP, which conduces to potentiating neuronal death [166,167,168]. Increased HC openings lead to neurotoxic molecule release, cell swelling, and increased coupling, which cause an increase in Ca^2+^ waves stressing astrocytes, until they can no longer protect neurons in such a toxic environment [169]. Additionally, ATP released by neurons and astrocytes activates Cx43 present in the astrocyte extremity of the BBB, which subsequently triggers intercellular Ca^2+^ changes in signaling and alters the biological properties of the BBB endothelial cells [170]. The release of ATP and glutamate is an important pathological manifestation and mechanism of neuroinflammation. This paragraph will describe the interrelationships in detail.

#### 5.4.1. ATP Release

**Figure 2:**
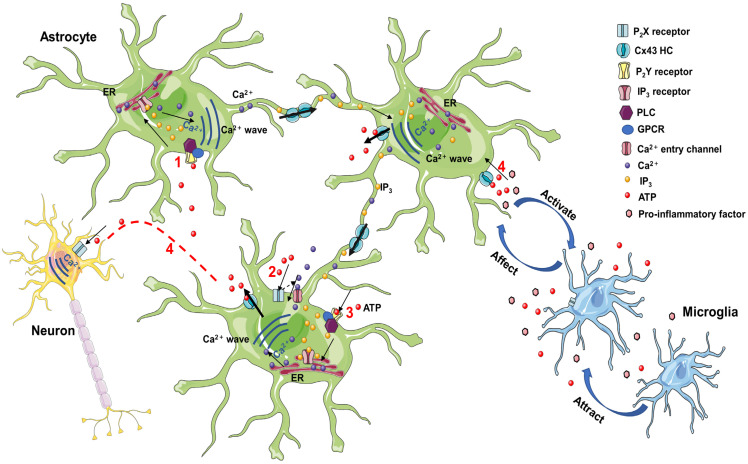
(1) Extracellular ATP stimulation to the activation of PLC and the formation of IP_3_, which, upon binding to ER receptors, promotes the release of Ca^2+^ stored in the ER. IP_3_ and Ca^2+^ diffuse to adjacent cells through GJC, resulting in the diffusion of intracellular Ca^2+^ waves. (2) Extracellular ATP leads to the entry of extracellular Ca^2+^ into the cell by activating membrane P_2_X receptors. (3) Activation of P_2_Y receptors by ATP leads to PLC activation and IP_3_ formation. Increased Ca^2+^ waves are induced by IP_3_ and P_2_X receptor opening, promoting ATP release through Cx43 HC, and extending Ca^2+^ waves to neighboring cells. (4) Astrocyte ATP release induces intraneuronal Ca^2+^ waves. Astrocyte HCs release ATP into the extracellular space, creating an ATP concentration gradient that triggers microglia activation. Extracellular ATP induces the microglia to release endogenous ATP that attracts distant microglia and promotes the inflammatory cascade. ATP, adenosine triphosphate; PLC, phospholipase C; IP_3_, 1,4,5-trisphosphate receptor; ER, endoplasmic reticulum; GPCR, G-protein-coupled receptors; GJC, gap junction channel; HC, hemichannel.

ATP-gated ionizing membrane, purinergic P_2_X_7_Rs, mediates the regulation of neuroinflammation in the CNS [171]. The P_2_X subfamily of ATP receptors is abundantly expressed on microglia, and P_2_X_7_ has been demonstrated to promote the pro-inflammatory cytokine maturation and secretion, modulating astrocytic responses to inflammation in the CNS [7]. Increased Cx43-dependent peptidoglycan expression results in ATP release that stimulates the microglial P_2_X receptor (P_2_XR) via an autocrine/paracrine loop, effectively exacerbating the production of pro-inflammatory mediators [172]. Additionally, astrocytes release ATP into extracellular space through the Cx43 HCs, establishing a concentration gradient of ATP and triggering microglial activation. Extracellular ATP induces microglia to release endogenous ATP that attracts distant microglia to the injury site, promoting the inflammatory cascade [171] (Figure 2).

There are two modes of propagation of Ca^2+^ waves between cells (Figure 2). One is the mediating role of intercellular transfer of 1,4,5-trisphosphate (IP_3_), which spreads through GJCs. Stimulation of receptors upstream of the cell leads to the activation of phospholipase C (PLC) to induce the formation of IP_3_, which activates the release of stored Ca^2+^ in the endoplasmic reticulum [173]. IP_3_ and Ca^2+^ diffuse to adjacent cells through GJCs, generating rising waves of intracellular Ca^2+^ concentrations [174]. The other is mediated by extracellular ATP as a paracrine messenger [175]. ATP activates P_2_ receptors. On the one hand, the stimulation of pro-metabolic P_2_Y receptor (P_2_YR) contributes to the activation of PLC and the formation of IP_3_, inducing an increase in intracellular Ca^2+^ [174]. On the other hand, the activation of pro-ionizing P_2_XR causes Ca^2+^ influx [174]. Co-action opens Cx43 HCs to promote ATP release. Increased ATP, released through HCs, leads to the activation of P_2_XR, while simultaneously activating metabotropic P_2_YR and triggering astrocyte Ca^2+^ signaling [176]. Physiological Ca^2+^ signaling sensitizes the opening of Cx43 HCs, extending Ca^2+^ waves to the neighboring cells [177]. ATP is degraded to adenosine outside the cell, which inhibits excitatory activity and presynaptic neurotransmitter release, thereby affecting neuronal function [178].

In organotypic spinal cord slices, diverse inflammatory threats induce Ca^2+^ release from the endoplasmic reticulum, mitochondria, pro-inflammatory activation of astrocytes, and production of cytokines and chemokines, tuned by Cx43 GJCs and Cx43 HCs [179]. P_2_XR activation mediates the evoking of NLRP_1_/NLRP_2_ inflammasomes in neurons/astrocytes, and NLRP_3_ inflammasomes in the microglia [27]. Similarly, ATP activates ionophilic P_2_X_7_R in endothelial cells, leading to the production of IL-1β, which may result from inflammasome activation after P_2_X_7_R activation [180,181]. IL-1β causes transient changes in P_2_X_7_R astrocyte expression, resulting in enhanced Ca^2+^ influx responses to specific P_2_X_7_R ligands [182]. Upon stimulation by inflammatory conditions, such as IL-1β or TNF-α, endothelial cells or astrocytes respond through intracellular signaling and ATP release through Cx43, possibly involving NF-κβ signaling associated with the induction of nitric oxide (NO) synthase activation and NO production [183].

#### 5.4.2. Glutamate–Glutamine Cycle

Abnormal glutamate dysfunction and glutamate circulatory system dysfunction exist in patients with depression [184]. Glutamate is an excitatory neurotransmitter implicated in the pathophysiological process of neuropsychiatric diseases, such as depression. Increased extracellular glutamate is detrimental to neurons, often associated with inflammation and stress. Glutamate is redistributed through GJCs in the astrocyte network and shuttled back to neurons as glutamine. Exposure of cerebral endothelial cells to excitotoxic levels of glutamate activates cell-expressed NMDAR, disrupting the BBB [170].

**Figure 3:**
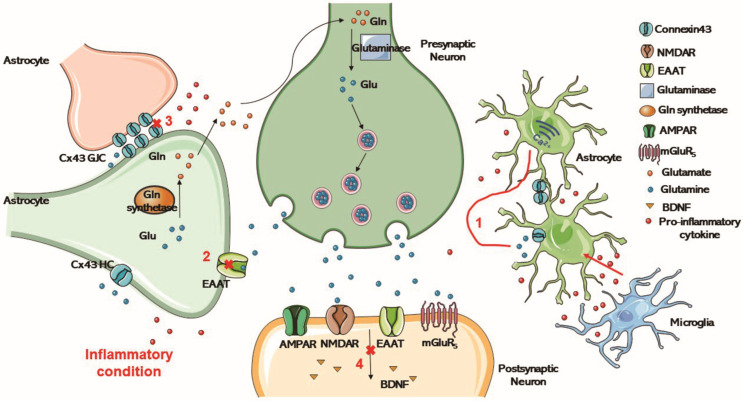
Glu is released by vesicles within presynaptic neurons upon excitation, and activates various ionotropic and metabotropic receptors on presynaptic and postsynaptic neurons and glial cells upon release. Astrocytes transport and clear glutamate through highly efficient EAAT. In the cytoplasm, glutamate is converted to Gln, which is exported and taken up by neurons and hydrolyzed to glutamate by glutaminase. (1) Inflammation in the brain limits the ability of the astrocytes to clear spilled glutamate. (2) Excessive glutamate release reduces the amount and function of EAAT. (3) In cells undergoing inflammation, glutamate release from the extracellular space is increased, inducing intercellular Ca^2+^ signaling and the down-regulation of Cx43 gap junctions. (4) The glutamate transporter is altered and the normal production of BDNF is affected. Glu, Glutamate; Gln, glutamine; HC, hemichannel; GJC, gap junction channels; EAAT, excitatory amino acid transporter; AMPAR, a-amino-3-hydroxy-5-methyl-4-isoxazolepropionic acid receptor; NMDAR, N-methyl-D-aspartate-receptor; mGluR_5_, metabotropic glutamate receptor 5; BDNF, brain-derived neurotrophic factor.

Glutamate is released by vesicles within presynaptic neurons upon excitation (Figure 3). Upon release, glutamate activates various ionizing and metabolic receptors on the presynaptic and postsynaptic neurons, as well as the glial cells [185]. Endothelial cells of the BBB take up glutamate and glutamine through transporters in their outer membranes, keeping low levels of the interstitial glutamate [138]. Astrocytes transport and clear glutamate via the efficient excitatory amino acid transporters (EAATs) and glutamate transporter-1 (GLT-1) [186,187]. In the cytoplasm, glutamate is converted to glutamine, which is exported and taken up by neurons and hydrolyzed to glutamate by glutaminase [185,188]. Glutamate accumulated in astrocytes is converted to glutamine by the action of glutamine synthetase, and glutamine is transported to neurons for recycling as a precursor of gamma amino butyric acid (GABA) and/or glutamate [175].

In cells undergoing inflammation, increased glutamate release from the extracellular space, downregulation of Na^+^/K^+^ ATPases, alterations in the glutamate transporters GLT-1 and glutamate–aspartate transporter (GLAST), and the reorganization of actin filaments in the cytoskeleton cause the down-regulation of intercellular Ca^2+^ signaling and Cx43 gap junctions [23]. Neuroactive-derived glutamate activates the metabolic glutamate receptors, which activate astrocytes, increasing intracellular Ca^2+^ [189]. LPS can be used to establish a cell inflammation model in vitro. Ca^2+^ transients from single peak to Ca^2+^ oscillations is evoked by glutamate when astrocytes are incubated with long-term LPS [176]. Intracellular Ca^2+^ is thus released from the endoplasmic reticulum increasingly, contributing to oscillatory changes in intracellular Ca^2+^ dependent on the overstimulation of metabotropic glutamate receptor 5 (GqmgluR_5_) [176]. In turn, Ca^2+^ waves spread across the astrocyte network. The glutamate transporter is altered, and the normal production of brain-derived neurotrophic factor (BDNF) is affected [6]. From the results of related experimental studies in recent years, it can be seen that inflammatory cytokines induce COX-2 production after triggering the p38MAPK/iNOS pathway, leading to the opening of Cx43 HCs, thereby releasing glutamate to activate NMDA receptors [190].

## 6. Conclusions and Perspectives

In this review, the possible mediating effects of Cx43 in neuroinflammation and depression is mainly expounded. In the activation of inflammatory pathways, inflammatory cytokines play an important role, which disrupts the normal function of endothelial cells, glial cells, and the function of Cx43. Inflammatory cytokines are closely related to every link to depression. Alongside that, the activation of inflammatory pathways in the brain will affect the release and uptake of glutamate and ATP in the synaptic cleft, hindering the effect of traditional antidepressant drugs. To the best of our knowledge, the underlying mechanism of depression, induced by Cx43 dysfunction and neuroinflammation, has not been revealed, and further study of Cx43 might contribute to revealing the pathogenesis of depression. Cx43 could be developed as a novel therapeutic target for depression.

## Figures and Tables

**Table 1:** Changes in Cx43 of astrocytes, and inflammatory signatures in different neuropsychiatric diseases.

Disease Types	Expression	Function	Pro-Inflammatory Cytokines	Anti-Inflammatory Cytokines	References
AD	Increase (Around Aβ)	Inhibition (GJCs) Promotion (HCs)	↑	↓	[29,30,31]
PD	Increase (In rodent striatum)	— —	↑	↓	[28]
MDD	Decrease	Inhibition (GJCs) Promotion (HCs)	↑	↑	[6]
Epilepsy	Increase (In hippocampus)	Inhibition (GJCs) Promotion (HCs)	↑	↓	[28,32,33,34]
Glioma	Increase (In the peri-tumor region)	Inhibition (GJCs) No significant increase (HCs)	↑	↓	[35,36,37]
Ischemic Stroke	No significant change	Inhibition (GJCs) Promotion (HCs)	↑	↓	[38,39]

AD, Alzheimer’s disease; PD, Parkinson’s Disease; MDD, major depressive disorder; Aβ, amyloid β-peptide; GJC, gap junction channel; HC, hemichannel; ↑, Increase; ↓, Decrease.

## Data Availability

The data presented in this study are available on request from the corresponding author.
